# Nestin expression in mesenchymal stromal cells: regulation by hypoxia and osteogenesis

**DOI:** 10.1186/s12917-014-0173-z

**Published:** 2014-08-05

**Authors:** Alice Wong, Ehssan Ghassemi, Clare E Yellowley

**Affiliations:** 1 Department of Anatomy, Physiology, and Cell Biology, School of Veterinary Medicine, University of California Davis, Davis 95616, CA, USA

**Keywords:** Nestin, Mesenchymal stromal cells, Canine, Equine, Human, Fracture callus, Osteogenesis, VEGF

## Abstract

**Background:**

The intermediate filament protein nestin is used as a marker for neural stem cells, and its expression is inversely correlated with cellular differentiation. More recently, nestin expression has also been described in other cell types including multipotential mesenchymal stromal cells (MSCs). In this study, we examined the expression of nestin in equine, canine and human bone marrow-derived MSCs undergoing osteogenic differentiation, to determine whether nestin levels were attenuated as the cells acquired a more mature phenotype. In addition, the expression of nestin may be under the influence of cellular hypoxia, as nestin expression is known to increase in areas of ischemic tissue damage. Therefore, we also examined the effects of hypoxia on expression of nestin in human MSCs and examined a role for hypoxia inducible factor 1-alpha (HIF-1α) and vascular endothelial growth factor (VEGF) in the response. Additionally, we quantified the temporal expression of nestin in the fracture callus during bone regeneration, a site that has been characterized as hypoxic.

**Results:**

There were no significant changes in nestin expression in MSCs during osteogenic differentiation. There was a significant increase in expression of nestin mRNA and protein in human MSCs in response to hypoxia (1% O_2_) or the chemical hypoxia mimetic desferroxamine. This may be due to upregulation of VEGF under hypoxia, as treatment of cells with the VEGF receptor antagonist CPO-P11 attenuated hypoxia-induced nestin expression. A significant increase in nestin mRNA expression was observed in the fracture callus of mice three and seven days post fracture.

**Conclusions:**

Nestin was not a selective marker for MSCs, as its expression was maintained during osteogenic differentiation, in all species examined. Furthermore our data suggest that nestin expression can be induced by hypoxia, and that this increase in nestin is partially regulated by HIF-1α and VEGF. Interestingly, nestin levels were significantly upregulated at the fracture site. Further studies are required to understand the role of nestin in bone cell biology and ultimately bone regeneration.

## Background

The multilineage potential of MSCs was first reported in 1966 by Friedenstein et al. [1]. Subsequently, numerous studies investigated the potential of MSCs to participate in the repair and regeneration of diseased and damaged tissue, including bone [2]–[4]. MSCs migrate to sites of tissue damage by recognizing and responding to chemokines critical for cell homing [5]–[8]. Indeed MSCs were shown to improve the biomechanical properties of regenerated callus in a mouse model [4]. Yet the intricate mechanisms dictating the activation, trafficking and beneficial properties of MSCs are still under investigation.

The isolation and definitive characterization of MSCs is complex. The number of MSCs found in adult tissue is relatively low and appears to decrease with age, so that cell-based therapies will likely rely on the expansion of MSC populations ex vivo [9]. In order to isolate and expand such cells to determine their roles in bone or other tissue regeneration, markers and methodology for distinguishing undifferentiated MSCs from a heterogeneous cell population must be clearly defined. Currently, MSCs are characterized by their ability to self-renew and undergo tri-lineage differentiation into osteoblasts, adipocytes, and chondrocytes; these cells are further identified as CD105+, CD73+, CD90+ cells that lack CD45, CD34, CD14 or CD11b, CD79alpha or CD19 and further lacking HLA-DR surface molecules [10],[11]. However these requirements may not be conserved between species [12].

Nestin, an intermediate filament, has been identified as a marker of neural stem cells [13]–[15]. The expression of nestin in these cells is inversely correlated with cellular differentiation and thus it is thought to represent a developmentally regulated marker of immature cell status [14]. Increases in the number of nestin-immunopositive cells have been reported in areas of the damaged rat brain following cerebral ischemia and traumatic brain injury [16]. However, nestin expression may not be limited to neuronal lineage cells and moreover may represent a common marker for multilineage progenitor cells [17]–[19]. Nestin expression has been demonstrated in human, plastic adherent bone marrow derived MSCs [18]. Additionally, CD45-/nestin + cells from bone marrow were able to form colony-forming unit fibroblasts, and were able to undergo tri-lineage differentiation [17].

Nestin expression may also be regulated by hypoxia. The bone marrow niche, which contains nestin-positive progenitor cells, is hypoxic [20] and nestin expression increases in cells near areas of ischemic damage in rat brain [21], kidneys [22] and heart [23]. In this study we examined the expression of nestin in equine, canine and human bone marrow derived MSCs undergoing osteogenic differentiation to determine whether nestin levels were attenuated as the cells acquired a more mature phenotype. We also examined the effects of hypoxia on expression of nestin in human MSCs and examined a role for hypoxia inducible factor 1-alpha (HIF-1α) and vascular endothelial growth factor (VEGF) in the response. Additionally, we quantified the temporal expression of nestin in the fracture callus during bone regeneration, a site that has been characterized as hypoxic [24]–[27].

## Results

### Nestin expression is maintained during MSC differentiation

**Figure 1:**
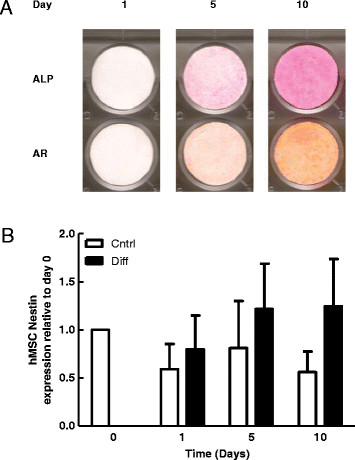
**Alkaline phosphatase activity, calcium deposition and nestin expression during osteogenic differentiation of human MSCs. A**. Representative wells demonstrating the effects of time in osteogenic media on alkaline phosphatase activity (ALP) and calcium deposition (Alizarin red staining; AR). **B**. Effects of time in osteogenic media on Nestin expression in hMSCs. Bars represent mean *nestin* expression normalized to *ribosomal protein L13* ± SEM relative to day 0 (n = 5).

**Figure 2:**
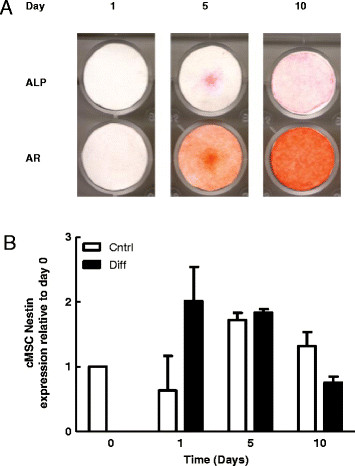
**Alkaline phosphatase activity, calcium deposition and nestin expression during osteogenic differentiation of canine MSCs. A**. Representative wells demonstrating the effects of time in osteogenic media on alkaline phosphatase activity (ALP) and calcium deposition (Alizarin red staining; AR). **B**. Effects of time in osteogenic media on Nestin expression in cMSCs. Bars represent mean *nestin* expression normalized to *β2-microglobulin* ± SEM relative to day 0 (n = 5).

**Figure 3:**
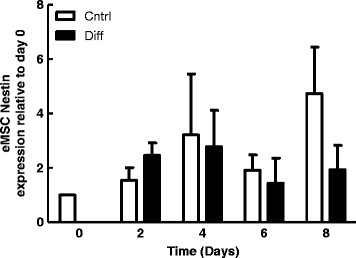
**Effects of time in osteogenic media on nestin expression in equine MSCs.** Bars represent mean *nestin* expression normalized to *PPIB* ± SEM relative to day 0 (n = 5).

An increase in ALP staining in parallel with positive AR staining was used as a qualitative evaluation of osteogenic differentiation. Increased ALP and AR staining over time in osteogenic media confirmed that both human and canine MSCs differentiated in culture (Figures 1 and 2, respectively). We have previously confirmed osteogenic differentiation of equine bone marrow-derived MSCs under conditions identical to those used here [28]. There were no significant changes in nestin expression in MSCs induced to undergo osteogenic differentiation, in any species investigated (Figures 1B, 2B and 3). Nestin mRNA expression was normalized across time points to day 0 expression. Our findings suggest that nestin expression is not correlated to osteogenic differentiation state.

### Hypoxia induced nestin expression in hMSCs

**Figure 4:**
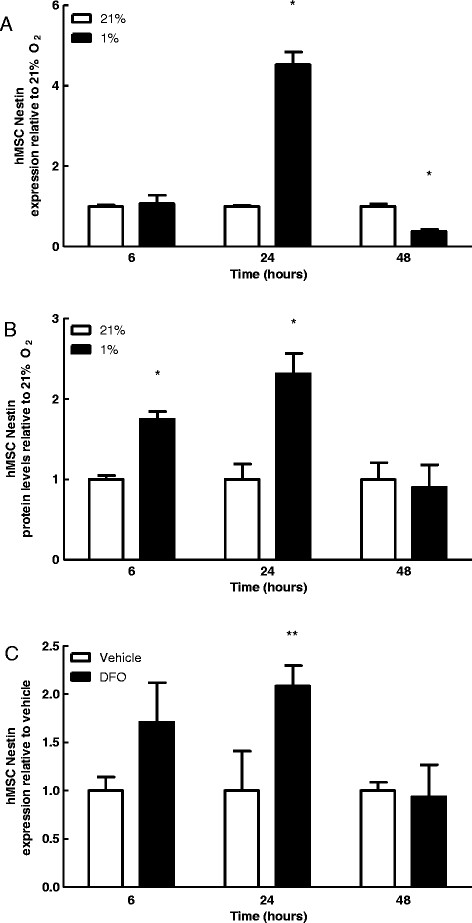
**Hypoxia and HIF-1α stabilization promote Nestin expression in hMSCs. A**: qPCR analysis of nestin expression after 6, 24 and 48 hrs of culture at 21% or 1% O_2_. Bars represent mean *nestin* expression normalized to *ribosomal protein L13* ± SEM relative to 21% at that same time point (n = 3). *Indicates p < 0.05. **B**: Quantification of nestin protein expression in hMSCs cultured in 21% and 1% O_2_. Bars represent mean protein expression normalized to tubulin ± SEM relative to 21% at the same time point (n = 2) **C**: qPCR analysis of Nestin expression after 6, 24 and 48 hrs of culture at 21% O2 in the presence of 100 μM desferroxamine (DFO), an iron chelator and stabilizer of HIF-1α (n = 3). *Indicates p < 0.05, **indicates p < 0.01.

There was a significant increase in nestin mRNA expression in hMSCs at 24 hours post incubation in 1% O_2_ compared to cells incubated at 21% O_2_ (Figure 4A). This rise in nestin expression was transient, as it was followed by a significant decrease at 48 hours (Figure 4A). Nestin protein levels were significantly greater in 1% O_2_ at both 6 and 24 hrs (Figure 4B). Furthermore, treatment of hMSCs with desferroxamine, an iron chelator and stabilizer of HIF-1α, increased nestin mRNA expression at 6 and 24 hours, at which time this increase achieved statistical significance (Figure 4C). Our data suggest that nestin mRNA and protein levels are increased under hypoxic conditions via a mechanism requiring the HIF-α family of transcription factors.

### hMSC nestin expression is inhibited following treatment with VEGF receptor antagonist

**Figure 5:**
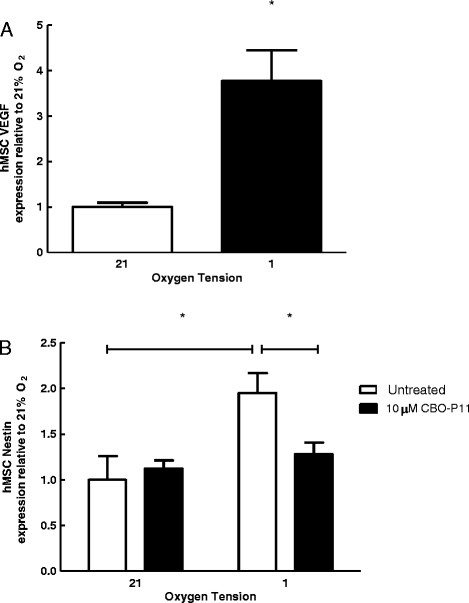
**Inhibition of nestin expression by VEGF receptor antagonist. A**. qPCR analysis of VEGF expression in hMSCs cultured at 21% or 1% O_2_ for 24 hrs. Bars represent mean VEGF expression normalized to *ribosomal protein L13* ± SEM expression relative to 21% O_2_ (n = 3). **B**. qPCR analysis of nestin expression after 24 hrs of culture at 21% and 1% O_2_ in the presence or absence of 10 μM of CBO-P11, a VEGF receptor antagonist. Treatment with CBO-P11 prevents the increase in Nestin observed in hypoxic conditions. Bars represent mean *nestin* expression normalized to *ribosomal protein L13* ± SEM expression relative to 21% O_2_ control (n = 3). *Indicates p < 0.05.

VEGF levels are increased under hypoxia via a mechanism that involves the HIF-α family [29],[30]. VEGF in turn has been shown to control the expression of a number of genes including annexin A2 [31],[32]. We observed a significant increase in VEGF expression in hMSCs cultured in 1% O_2_ for 24 hrs when compared to cells cultured at 21% O_2_ (Figure 5a). Interestingly, treatment of cells with the VEGF receptor antagonist CPO-P11 attenuated 1% O_2−_induced nestin expression at 24 hrs (Figure 5b). Our data suggest that VEGF plays a role in the mechanism by which nestin expression increases under hypoxic conditions.

### Nestin expression increases over time in the fracture callus

**Figure 6:**
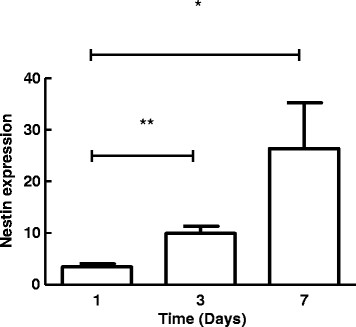
**Nestin expression increases over time in the fracture callus. qPCR analysis of nestin expression in the murine fracture callus.** Bars represent mean expression ± SEM (n = 3). *Indicates p < 0.05. **Indicates p < 0.01.

A significant increase in nestin mRNA expression was observed in the fracture callus of mice three and seven days post fracture (Figure 6).

## Discussion

Nestin is recognized as a marker of neural progenitor cells, wherein nestin expression is inversely correlated with cellular differentiation and is thus developmentally regulated [14],[19]. Specifically the transition from proliferating neural stem cell to post-mitotic neurons leads to the down-regulation of nestin [19],[33]. Recent studies have indicated that nestin is also expressed in a variety of other cells including cells of mesenchymal phenotype, hair follicle stem cells [34] and newly proliferating endothelial cells [35], and may be a potential general marker of immature cell types [18],[34]–[36]. Specifically CD45^−^nestin^+^ cells have been described as MSCs, maintaining self-renewal and multilineage mesenchymal differentiation potential [17]. However, depending on cell source nestin expression in mesenchymal-like stem cells can be variable [37]. In our study nestin was expressed in MSCs derived from equine, canine and human bone marrow. Nestin mRNA expression did not significantly change during osteogenic differentiation. The maintenance of nestin levels throughout the process of osteogenic differentiation suggests that nestin expression is not exclusive to undifferentiated MSCs, but is maintained throughout their osteogenic lineage commitment and differentiation. The consistency of this finding between between species suggests that nestin expression is not an effective means of determining the unspecialized status of MSCs.

Nestin expression levels are increased in tissues with ischemic damage [21]–[23]. Increased nestin at sites of ischemia could be due to the migration of nestin positive progenitors to the site, or up-regulation of nestin expression in resident progenitors or more committed cells in response to the hypoxic environment. Nestin expression has been shown to be enhanced in astrocytes exposed to hypoxia and down regulated following hyperoxia [38]. Our data show that there was a significant increase in nestin protein expression in hMSCs as early as 6 h after exposure to 1% O_2_ compared to cells incubated at 21% O_2_. Hypoxia is known to upregulate genes via the hypoxia-inducible factor (HIF) transcription pathway. Under normoxic conditions HIF-1α is hydroxylated by prolyl hydroxylase-domain proteins (PHDs), ubiquitinated by the von Hippel-Lindau tumor suppressor protein, and undergoes proteosomal degradation. However, under hypoxic conditions the activity of PHDs is reduced, HIF-1α accumulates and forms a heterodimer with HIF1-β. The HIF-1 complex translocates to the nucleus and binds to the hypoxia-responsive element (HRE) on target genes to activate transcription [39],[40]. To determine the role of HIF-1α in hypoxia-induced nestin expression we used DFO. DFO chelates iron, an essential co-factor for the function of prolyl-hydroxylase domains (PHDs), which under normoxic conditions leads to stabilization and accumulation of HIF-1α [41],[42]. In our study, DFO increased nestin mRNA expression at 6 hrs that became significantly increased over control at 24 hrs. Our data show that nestin levels are increased under hypoxic conditions, via a mechanism requiring the HIF-1 family of transcription factors.

Vascular endothelial growth factor (VEGF) plays a pivotal role in neovascularization; and stimulates angiogenesis necessary for endochondral ossification [43],[44]. VEGF is induced by hypoxia under the control of HIF-1α [30]. Interestingly, we and others have shown that proteins such as annexin A2 are upregulated under hypoxic conditions in a process that requires VEGF [31],[32]. Specifically we showed that addition of a VEGF inhibiting antibody or CBO-P11, a VEGF receptor antagonist, to cells cultured under hypoxia for 24 hours attenuated induction of annexin A2. In this study, as expected, we observed a significant increase in VEGF expression in hMSCs cultured in 1% O_2_, and treatment of cells with CBO-P11 attenuated hypoxia-induced nestin expression. This suggests that VEGF plays a significant role in the mechanism by which nestin expression increases under hypoxic conditions.

Areas of bone damage, such as fracture are hypoxic in nature due to disruption of the vasculature find [24]–[27]. MSCs are known to home to, and proliferate in areas of tissue injury and play a distinctive role in the healing process including neovascularization and formation of a fracture callus [45],[46]. Thus it is likely that the arrival of nestin expressing MSCs, and/or the hypoxic nature of the fracture site might result in increased nestin levels during bone healing. Our study demonstrated a significant increase in nestin mRNA expression in a callus at days three and seven days post femoral fracture in the mouse. The role of nestin during bone healing is not known. A decrease in nestin expression has been shown as an essential prerequisite for the induction of Cdk5-dependant apoptosis in neural progenitor cells [47]. Nestin was also shown to play a protective role against high glucose induced apoptosis in murine podocytes [48]. The central role of nestin may therefore be to protect cells from apoptosis in a hypoxic environment and maintain cellular proliferative status in areas of tissue injury.

## Conclusion

In summary, nestin was expressed in undifferentiated MSCs of human, canine and equine origin. Furthermore, contrary to our hypothesis that nestin is a marker of undifferentiated MSCs, nestin expression was maintained throughout their lineage commitment and osteogenic differentiation. Our data also show that nestin expression is significantly upregulated by hypoxia and that this increase in nestin is in part regulated by HIF-1α and VEGF. Interestingly nestin levels were significantly upregulated at the fracture site, an environment rendered hypoxic by disruption of blood vessels. Further studies are required to understand the role of nestin in bone cell biology and ultimately bone regeneration.

## Methods

### Cell culture

Canine MSCs (cMSCs) were isolated from bone marrow as described [49]. Equine MSCs (eMSCs) were derived from bone marrow isolated from Quarter horses as described [28]. Bone marrow was acquired with the approval from the Institutional Animal Care and Use Committee of University of California, Davis, USA. Briefly, equal volume of HBSS was added to the bone marrow aspirate and centrifuged for 15 min at 300 × g. After removing the supernatant, the pellet was re-suspended in HBSS and centrifuged once more for 5 min at 1000 × g. The pellet was then re-suspended in media and plated. Adult human bone marrow-derived MSCs (hMSCs) were purchased from Lonza and used as approved by the UC Davis Institutional Biological Committee (880-02B). Cells were seeded at 5,000 cells/cm^2^ in growth media (RPMI for cMSCs and eMSCs, and α-MEM for hMSCs), supplemented with 10% fetal bovine serum, and 1% penicillin-streptomycin) and incubated in a standard humidified incubator (HERAcell 150, Thermo Fisher, USA) at 21% O_2_, 5% CO_2_, and 37°C. Growth media was replaced every 3 days and cultures were passaged upon reaching 70-80% confluence. Canine cells were used from passages 2 to 4, while equine and human cells were used from passages 3 to 5.

### Osteogenic differentiation

MSCs from canine, human and equine sources were seeded at 3000 cells/cm^2^ in 6-well plates in growth media. Twenty-four hours after seeding, RNA samples were collected (day 0) and growth media was replaced with osteogenic media (growth media supplemented with 100 nm dexamethasone, 10 mM β-glycerophosphate and 50 μg/mL ascorbic acid phosphate). RNA samples were collected at days 0, 1, 5 and 10 for hMSC and cMSC studies, while RNA samples for eMSC studies were collected on days 0, 2, 4, 6 and 8.

### Alizarin Red staining

On days 1, 5, and 10, hMSC and cMSCs were washed with pre-warmed PBS (37°C) and fixed for 15 min with 5% paraformaldehyde solution. After fixing, cells were washed with water and incubated with 0.5% Alizarin Red S (AR; Sigma Aldrich) solution for 15 min. The solution was then removed and the cells were washed with water until the wash water was clear. Plates were allowed to air-dry prior to imaging. Stained samples were scanned using a Canon flatbed scanner. AR staining of differentiating eMSCs was previously performed and described [28].

### Alkaline phosphatase staining

On days 1, 5, and 10, hMSC and cMSCs were stained for alkaline phosphatase (ALP) using a commercially available kit (Sigma-Aldrich). Briefly, cells were washed with pre-warmed PBS (37°C) and fixed with citrate-acetone-formaldehyde solution. After fixing, cells were washed with water, incubated with alkaline dye mixture for 15 min, washed and air dried. Cells were then scanned using a Canon flatbed scanner. ALP staining of differentiating eMSCs was previously performed and described [28].

### Hypoxia studies

Cells were plated at 5,000 cells/cm^2^ in 12-well plates and 24 h later media was replaced with pre-conditioned media from each oxygen tension (21% or 1%). For ambient (21%) oxygen tension, cells were then cultured in a standard humidified incubator at 37°C with 5% CO_2_. For 1% oxygen tension experiments, cells were cultured in humidified incubators at 37°C with 5% CO_2_ with oxygen tension reduced to 1% using supplemental nitrogen (Heracell 150, Thermo Fisher USA) for the indicated time. A subset of cells were treated with 100 uM desferrioxamine (Sigma, USA), an iron chelator known to stabilize HIF-1α under normoxic conditions. In addition, nestin expression was analyzed after treatment with 10 μM vascular endothelial growth factor (VEGF) inhibitor CBO-P11 (EMD Millipore).

### Realtime qPCR quantitative real-time PCR (qPCR)

RNA was purified using the RNeasy Mini Kit (Qiagen) and reverse transcribed using the Quantitect Reverse Transcription Kit (Qiagen). Mouse fracture samples were obtained from a previous study [50]. qPCR for mouse and human samples was performed using the QuantiFast PCR Master Mix (Qiagen), and TaqMan primer/probes (LifeTechnologies), the assay numbers are listed in Table 1. PCR products were amplified under the following conditions: 95°C for 3 min, followed by 40 cycles at 95°C for 3 s and 60°C for 30s. QuantiFast SYBR Green PCR Master Mix (Qiagen) was used to amplify canine and equine genes, the primers are listed in Table 2. Amplification was performed under the following conditions: denaturation at 95°C for 5 min, 40 cycles of denaturation at 95°C for 10 s, annealing at 60°C for 30s, followed by a melting curve procedure. Gene expression was normalized to the loading control (ΔC_t_) [51], and was further normalized to control conditions.

**Table 1:** Taqman qPCR targets and assay numbers

**Target**	**Assay no.**	**Species**
*nestin*	Hs04187831_g1	Human
*VEGF*	Hs00900055_m1	Human
*ribosomal protein L13*	Hs00744303_s1	Human
*nestin*	Mm00450205_m1	Mouse
*beta-actin*	Mm00607939_s1	Mouse

**Table 2:** SYBR green qPCR targets and primers

**Target**	**Genbank**	**Species**	**Forward**	**Reverse**
*nestin*	XM_547531	Canine	TCCAGAAGAGCTTGGTAAGG	CTGGGTGGCAGACAGTGGCA
*β2-microglobulin*	[30]	Canine	TCTACATTGGGCACTGTGTCAC	TGAAGAGTTCAGGTCTGACCAAG
*nestin*	XM_001915709	Equine	ACTGAGAAGTTCCAGCTGGC	TCAGCCTCTAGAAGGGTCC
*PPIB*	NM_001099761	Equine	GCTCTGTCTTCTTCCTGCTGTTG	CCAATTCGCAGGTCAAAGTACA

### Western blot

Cellular lysates were prepared in RIPA buffer (0.1% Triton X-100, 10 mM Tris, pH 8, 1 mM EDTA, 200 nM Na_3_VO_4_) with HALT protease inhibitors (Pierce). Protein concentration was determined using the DC Protein Assay (Biorad) following the manufacturer’s instructions. Samples (40 g protein) were run on a 10% SDS gel and transferred to a nitrocellulose membrane. The membrane was probed with nestin (1:100; Novus) or tubulin (1:1000; Cell Signaling) antibody overnight at 4°C, followed by the appropriate HRP-conjugated secondary antibody (1:1,000; Jackson ImmunoResearch), and developed with enhanced chemiluminescent substrate (Denville). Densitometric analysis was performed with the VisionWorks analysis software (UVP).

### Fracture callus

Murine fractures were performed as previously described [50]. Briefly, transverse closed femoral fractures were created in 13- to 14- week old, male C57BL/6 mice, whose femurs had been stabilized with a stainless steel pin prior to fracture. The contralateral femurs were used as controls.

RNA was isolated from the fracture callus as previously described [50]. Briefly, the callus was cut from the femur, flash frozen in liquid nitrogen and processed with TRIzol (Invitrogen). Total RNA was purified using RNeasy Mini Kit (Qiagen) and reverse transcribed to cDNA with Quantitect Reverse Transcriptase Kit and qPCR performed using the methods described above. Results were normalized to the loading control to yield ΔC_t_, then normalized to the contralateral limb to yield ΔΔC_t_[51]. All procedures were approved by the Institutional Animal Care and Use Committee of the University of California, Davis.

### Statistical analysis

Data were presented as means and error bars represent standard error of the mean. Statistical analysis of means was performed using one-way and two-way ANOVA. A probability level of p < 0.05 was considered statistically significant.

## Abbreviations

MSCs: Mesenchymal stem/stromal cells

HIF-1α: Hypoxia-inducible factor 1alpha

cMSCs: Canine mesenchymal stem/stromal cells

eMSCs: Equine mesenchymal stem/stromal cells

hMSCs: Human mesenchymal stem/stromal cells

VEGF: Vascular endothelial growth factor

AR: Alizarin Red

ALP: Alkaline phosphatase

qPCR: Quantitative real-time PCR

## Competing interests

The authors declare that they have no competing interests.

## Authors’ contributions

CY conceived and designed the experiments. EG and AW performed the experiments and analyzed the data. CY, EG and AW wrote the paper. All authors have read and approved the final manuscript.
